# Clinical Remarks. Prof. Thomas F. Rochester, on Pneumonia

**Published:** 1871-02

**Authors:** F. Bradnack

**Affiliations:** Member of the Class


### ART. VI.—*Clinical Remarks.* PROF. THOMAS F. ROCHESTER *on Pneumonia.*

REPORTED BY F. BRADNACK, MEMBER OF THE CLASS.

Pneumonia generally first attacks the right lung, its lower lobe. From this position, (which may be regarded as a *point d'appui,*) the disease may travel upwards. It may sometimes be arrested by the interlobar fissures, which separations, it, however, often over-leaps. Thus it happens that we find the disease in different stages in the different lobes of the same lung; indeed, pneumonia generally pursues this course. Prof. Alonzo Clark, of New York, together with many other eminent observers, regard gray hepatization as a necessary restorative sequence of red hepitization. My own observations compel me to dissent from this exclusive view. In the first stage of this disease there occurs but little expectoration. But subsequently the characteristic rusty, or plum juice sputa is observed. I believe that this is a distinct secretion from the *air vesicles.* So regarded, we are enabled to understand the succeeding stages. The vesicles becoming occluded, there ensues carnification of the lung simultaneously. While maintaining this point, I fully admit that there exists inflammation outside the air passages, whence fluid is exuded into the parenchyma of the lung. The patients you have just seen in the wards, and who have so rapidly convalesced from pneumonia, had nothing but inflammation of the air vesicles (vesiculitis.) But, while admitting that inflammation frequently exists outside the air passages, I also allow that consolidation of lung may ensue, where there is no vesiculitis. But this form of the affection is rare. With it pleurisy is more apt to be associated. In these cases we have high febrile movement and dyspnœa, together with various constitutional symptoms of great severity. The respiratory murmur undergoes a gradual diminishing; and the dyspnœa becomes more marked, and yet the patient may go three or four weeks without coughing or expectorating. This is extravesicular pneumonia, in which there is exudation outside the air passages.

Simple, frank pneumonia is not difficult of recognition. First, there is the paroxysmal cough, followed by the rusty sputa. Supposing the disease has attacked the lower lobes of the right lung, percussion elicits a slight degree of dullness. In practicing percussion in this region, there is some danger of arriving at a partially erroneous diagnosis by percussing the subjacent border of the liver, and mistaking the sounds for those indicative of hepatization of the pulmonary tissue. It is very necessary that the possibility of this fallacy should be borne in mind. In the first stage, auscultation gives the crepitant rale. This is the positive sign of the existence of the disease. This rale is heard in the last half of the inspiratory, and in the first part of the expiratory, acts. More than anything else, it resembles the sound produced by rubbing between the fingers some thick solution of gum arabic. It is caused by the passage of the air separating the tenacious material which lines the inflamed surfaces of the vessicles. To Dr. Carr, of Canandaigua, N. Y., belongs the credit of having first accurately explained one of the modes of the causation of the crepitant rale.

Following this stage, bronchial respiration, and bronchial voice, often occur. These conditions indicate hepatization of the lung. At this stage there is usually a marked increase of the general symptoms. We may now get the crepitant rale, but it will probably be in the upper part of the lobe, or in the superjacent lobe; or, it may possibly occur in the other lung. The disease may now pass to the third stage, (gray hepatization,) or it may be resolved, mucous expectoration ensuing. The supervention of gray hepatization is generally a step towards health. The secretion is now muco-purulent. The condition of the patient improves, although dullness is still elicited on percussion. Auscultation likewise once more gives the crepitant rale, but this time it is the *rale redux*; and this is always an evidence of returning health.

It not infrequently happens that the third stage of pneumonia prevails in the lower lobe; the second stage in the middle, and the first stage in the upper lobe. The lobe first attacked is generally the first to recover. By reason of the simultaneous existence of the three stages of the disease, it is not uncommon for the characteristic rusty sputa to be mixed with a muco-purulent expectoration.

Now, what is the treatment of acute simple pneumonia? Since the time of my pupilage the treatment has undergone a very great change. Patients in those days were invariably treated to tartar emetic, and in no unstinting doses. In this disease there is a great tolerance of this heroic remedy. Notwithstanding I have known many patients to recover on this treatment, I would not, for my life, practice it now. Contrary to the opinion of many, I dissent from the view that, of late years, both patients and diseases have undergone great changes. It is not impossible that you may meet cases which would be benefited by the use of tartar emetic and venesection; but, as a general rule, the majority of cases will do vastly better without any depletory treatment. Old persons always bear it badly. Allow your patients all the cold water they desire to drink. Give as early as possible carbonate of ammonia. It is scarcely ever necessary to apply either cups or leeches. When pneumonia is complicated by malaria, quinia is rationally indicated.

Regarding the administration of opium, there is no disease in which more care is required in the use of this drug. Inasmuch as a strong predisposition to cyanosis exists, the employment of any measures which might tend to superinduce this state, is, of course, contradicted. Yet, many practitioners are so wedded to routine practice, that they find it almost impossible to treat this disease without opium. They frequently push this treatment too far. Opium *is* very useful, however, when judiciously employed.

In the treatment of pneumonia, very often but little medication is required. Diluents may be largely given. Frequently active stimulation is necessitated from the outset. Other cases will require a treatment diametrically opposite to this. If properly managed, a large proportion of cases of pneumonia eventuate in recovery.

